# Inhibitors of the Hepatitis C Virus RNA-Dependent RNA Polymerase NS5B

**DOI:** 10.3390/v2102169

**Published:** 2010-09-28

**Authors:** Megan H. Powdrill, Jean A. Bernatchez, Matthias Götte

**Affiliations:** 1 McGill University, Department of Microbiology and Immunology, 3775 University Room D6, Montreal Quebec, H3A2B4, Canada; E-Mail: megan.powdrill@mail.mcgill.ca; 2 McGill University, Department of Biochemistry, 3775 University Room D6, Montreal Quebec, H3A2B4, Canada; E-Mail: jean.bernatchez@mail.mcgill.ca

**Keywords:** HCV NS5B polymerase inhibitors, drug resistance, viral fitness, genetic barrier

## Abstract

More than 20 years after the identification of the hepatitis C virus (HCV) as a novel human pathogen, the only approved treatment remains a combination of pegylated interferon-α and ribavirin. This rather non-specific therapy is associated with severe side effects and by far not everyone benefits from treatment. Recently, progress has been made in the development of specifically targeted antiviral therapy for HCV (STAT-C). A major target for such direct acting antivirals (DAAs) is the HCV RNA-dependent RNA polymerase or non-structural protein 5B (NS5B), which is essential for viral replication. This review will examine the current state of development of inhibitors targeting the polymerase and issues such as the emergence of antiviral resistance during treatment, as well as strategies to address this problem.

## 1. Introduction

With over 170 million people infected worldwide, the hepatitis C virus (HCV) represents a significant public health burden [1]. The therapy currently available to those infected with HCV is a combination of ribavirin and pegylated IFN-α. Unfortunately, this treatment is ineffective in many individuals and is associated with adverse side effects [2]. Progress has been made recently in the development of new therapies with improved potency and tolerance in patients [3,4]. The enormous genetic variability of the positive-strand RNA virus, however, has proven to be a considerable obstacle in developing broadly effective antivirals. HCV can be divided into at least six major genotypes that differ by 31–33% at the nucleotide level and is further categorized into subtypes that differ in nucleotide sequence between 20–25% [5]. Individuals infected with genotype 1, *i.e.* the most prevalent in North America and Europe, represent a “difficult-to-treat” population that respond poorly to treatment with ribavirin and pegylated IFN-α. Sustained virologic response (SVR), *i.e.* no detectable HCV after 48 weeks of treatment, is less frequently observed within this group of patients [6–8]. Furthermore, within each infected individual, HCV exists as a heterogeneous population of different viruses, referred to as quasispecies [9,10]. Genetic diversity within the HCV genome arises due to a combination of factors. The HCV RNA-dependent RNA polymerase (RdRp) NS5B is error-prone and lacks proof-reading activity. Estimates of the mutation rate vary from 10^−3^ base substitutions per site per year, determined through analysis of genomes from infected patients and chimpanzees, to 10^−4^–10^−5^ substitutions per nucleotide per round of genome replication, based on mathematical modeling and comparisons with related viruses [10–12]. Like the current standard of care, direct-acting antivirals can show a broad spectrum of activities depending on the genotype. Moreover, the release of an estimated 10^12^ virus particles per day creates an extremely large, divergent group of genomes from which resistant variants can emerge and dominate the virus population under selective drug pressure [13]. The selection of resistance to different classes of direct-acting antivirals is well documented *in vivo* and *in vitro* [14,15]. Drug discovery and development efforts have focused mainly on the viral NS3 protease and the NS5B polymerase, though progress is rapidly being made with other viral and cellular targets. This review will focus on NS5B polymerase inhibitors, with an attempt to bridge clinical and biochemical findings.

## 2. Structure and Function of HCV NS5B

As described for many other viral polymerases, the structure of NS5B resembles a right hand and is divided into three subdomains, referred to as the palm, fingers and thumb. The palm domain contains the enzyme active site catalytic residues, including the highly conserved GDD motif [16,17]. The aspartic acid residues are involved in the nucleotidyl transfer reaction during polymerization. They bind two divalent metal ions, which in turn facilitate the nucleophilic attack of the 3’-hydroxyl group of the primer terminus or priming nucleotide on the α-phosphate of the incoming nucleotide. The metal ions may also be involved in the release of the pyrophosphate (PPi) product [18]. The structure of the free enzyme and HCV NS5B with a short RNA substrate suggests that the enzyme needs to undergo significant conformational changes to accommodate newly synthesized double-stranded RNA [16,19–21]. Structures of ternary complexes composed of HCV NS5B, an RNA template, and specifically bound nucleotide(s) remain to be determined.

The HCV NS5B polymerase is capable of initiating RNA synthesis *de novo*, *i.e.* in the absence of a primer, which is believed to be the mechanism of initiation *in vivo* [22–26]. Here, the positive-strand RNA serves as a template for synthesis of the intermediate minus-strand, which in turn serves as template for (+)–strand production. The initiation phase requires the polymerase to catalyze the formation of a phosphodiester bond between two bound nucleotides, referred to as priming and initiating nucleotides. In the rate-limiting step that follows, the newly formed dinucleotide is used as a primer for the addition of the third nucleotide, whereupon the enzyme switches to the elongation mode which renders the reaction highly processive and enzyme dissociation is extremely slow [27–29]. Small molecule inhibitors of NS5B may interfere with the functions of the enzyme at each of the aforementioned individual steps that are associated with distinct structures of the replicating complex.

## 3. Classes of Inhibitors Targeting NS5B

**Figure 1:**
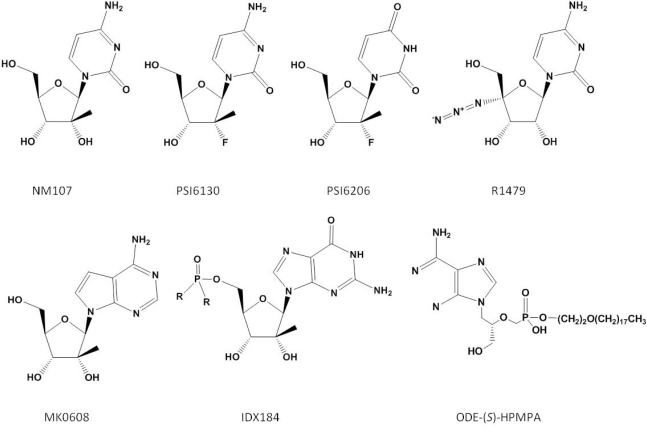
HCV inhibitors targeting the enzyme active site.

Three specific classes of inhibitors that target the polymerase have been reported. These include nucleoside analogue inhibitors (NIs), non-nucleoside analogue inhibitors (NNIs) and pyrophosphate (PPi) analogues. NIs bind at the enzyme active site and compete with their natural NTP counterparts for incorporation. Structures of the NIs discussed in this review are shown in Figure 1.

**Figure 2:**
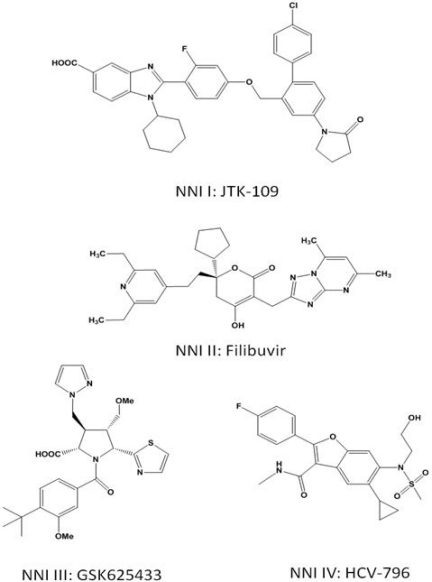
Structures of HCV NNIs binding at NNI sites 1 through 4.

These inhibitors can theoretically interfere with any step during RNA synthesis, provided there is appropriate base-pairing with the templated nucleotide. In contrast, NNIs bind at allosteric sites, and accumulating evidence suggests that these compounds inhibit polymerase activity specifically during initiation [30–32]. The structures of NNIs from each of the four allosteric binding sites are shown in Figure 2.

**Figure 3:**
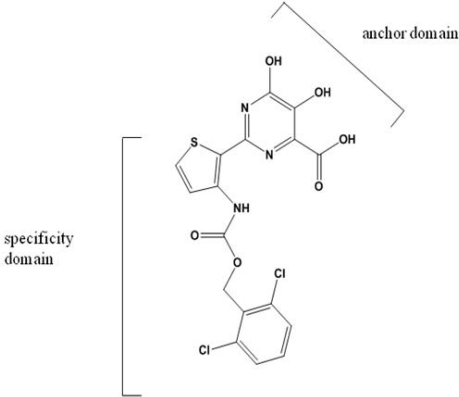
Structure of a PPi analogue. The inhibitor contains two distinct domains: the “anchor domain”, which is implicated in interactions with the two catalytic metal ions at the active site, and the “specificity domain”, which provides additional contacts at distant regions of the enzyme.

NIs and NNIs are currently being assessed in clinical trials. Investigational PPi analogues mimic the natural PPi released during the nucleotidyl transfer reaction. These molecules are designed to bind with an anchor domain to the PPi binding site (Figure 3) [33].

### 3.1. Nucleoside Analogues

This class of inhibitors has been described extensively for clinical use against hepatitis B virus, herpes viruses, and HIV [34,35]. The ionic nature of the phosphate moiety of nucleotide analogue inhibitors prevents their efficient permeation of the cellular membrane, which necessitates their delivery as uncharged nucleosides or as prodrugs. These are eventually phosphorylated to a 5′-triphosphate form by cellular kinases, with conversion to the monophosphate reported as the rate-limiting step [36,37]. The efficacy of NIs is dependent on a number of factors, including their capacity to be phosphorylated to the triphosphate form, how well they are incorporated by NS5B and their effectiveness as chain terminators. In contrast to most NIs that inhibit viral DNA polymerases, NS5B inhibitors still contain the 3′-hydroxyl group that, in theory, permits further nucleotide additions. However, binding and/or incorporation of the next nucleotide may be prevented through steric problems that literally cause termination. Therefore, these compounds are referred to as non-obligate chain terminators.

Biochemical experiments have shown that chain termination is not irreversible, and the presence of PPi at physiologically relevant concentrations can excise the incorporated inhibitor [38,39]. Thus, it is possible that excision of NIs may lower potency, although this has yet to be confirmed in cell-based assays or *in vivo* [38]. For HIV, it has been demonstrated that specific resistance conferring mutations can increase rates of excision of certain NIs [40]. In contrast, mutations associated with decreased susceptibility for HCV NIs do not show this phenotype.

Ribonucleotides that lack a 3′ hydroxyl group and prevent incorporation of the next nucleotide are not efficiently phosphorylated in cells and consequently have not been developed for use against HCV [41]. Non-obligate chain terminators that block HCV NS5B are 2′- or 4′-modified at the sugar moiety. 2′-modified compounds interfere with binding of the next nucleotide, while 4′-modified nucleotides appear to act as chain-terminators or as base pair confounders, *i.e.* they are incorporated into newly synthesized RNA but do not permit nucleotide incorporation during synthesis of the complementary strand [42–44]. Progress with regards to the clinical development of HCV NIs and NNIs, as reported on the NIH clinical trials website (http://clinicaltrials.gov), is summarized in Table 1 below.

Several structurally distinct NIs have advanced into the clinic. 2′-*C*-methylcytidine (NM107) is active against HCV in addition to a number of other (+)-single-stranded RNA viruses including bovine viral diarrhea virus, West Nile, dengue-2 and yellow fever virus [45]. The inhibitor acts as a non-obligate chain terminator; once incorporated it inhibits further nucleotide binding via a steric clash of the 2′-methyl group of the inhibitor with the incoming nucleotide [46]. The prodrug of 2′-*C*-methylcytidine (NM283, Valopicitabine) advanced to phase IIb clinical trials [47]; however, toxicity issues and insignificant improvement with regards to treatment outcome when combined with the standard of care have caused the development of NM283 to be placed on hold.

**Figure 4:**
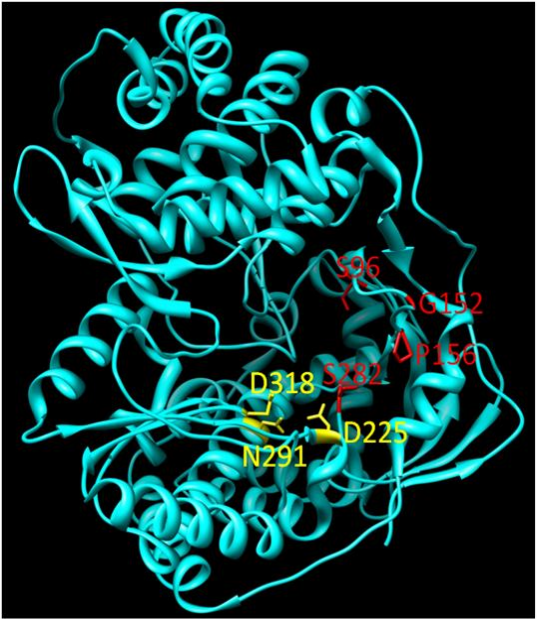
Positioning of mutations conferring resistance to HCV NI and PPi analogue inhibitors. Active site residues are highlighted in yellow and positions for resistance mutations in red. Mutations at the S96 and S282 positions confer resistance to NIs, while substitutions of the amino acids P156 and G152 leads to resistance to PPi analogues.

The related 2′-*C*-methyl-7-deaza-adenosine (MK0608) is also effective against a broad panel of (+)-ssRNA viruses [48]. Testing of the compound was undertaken in chimpanzees. MK0608 was effective against both genotype 1a and 3a infection, consistent with the concept that NIs are effective across genotypes due to the high level of conservation of the active site. Oral dosing of the inhibitor for 37 days in two chimpanzees led to a decline of −4.6log_10_ in one chimpanzee and a viral load below the limit of quantification in the second. After dosing, viral rebound occurred in both chimpanzees treated with the inhibitor. Sequencing of viral RNA at this point detected the S282T mutation [49]. This mutation is positioned at the enzyme active site (Figure 4) and is associated with resistance to 2′-*C*-methylated compounds via a steric clash with the methyl groups of the inhibitor and the methyl group of the substituted threonine residue [50].

The prodrug of 2′-methylguanosine (IDX184) has also entered clinical trials. Three day treatment of genotype 1, treatment-naïve patients with the compound resulted in −0.47log_10_ to −0.74log_10_ HCV RNA reductions with no serious side effects reported [51]. In addition, combining this analogue with an NNI or protease inhibitor was effective in suppressing the emergence of viral resistance [52]. Studies are now underway to evaluate the inhibitor in combination with pegylated IFN-α and ribavirin in HCV infected patients.

β-d-2′-deoxy-2′-fluoro-2′-C-methylcytidine (PSI6130) likewise acts as a non-obligate chain terminator [53]. Studies on the metabolic activation of this compound to its 5′ triphosphate form found that it can serve as a substrate for a deaminase, leading to the presence of both PSI6130 and its uridine counterpart [54]. Treatment with PSI6130, therefore, potentially presents two distinct species to target NS5B. The uridine analogue, PSI6206 (PSI7851/PSI7977 chirally pure form), shows specific activity against the NS5B polymerase, with no activity towards closely related members of the *Flaviviridae* family such as West Nile or yellow fever virus [55]. PSI6206, however, appears to be inefficiently phosphorylated to its monophosphate form in cells [56]. Delivery as a phosphoramidate prodrug has overcome this limitation [56]. Three day treatment with PSI7851 of treatment naïve, genotype 1 patients saw up to a −1.95log_10_ reduction in HCV RNA [57]. PSI7977 is currently in phase IIa trials. The prodrug of the cytidine analogue (R7128) is also currently undergoing trials evaluating R7128 in combination with pegylated IFN-α and ribavirin.

Several more recent trials have been designed to assess the efficiency of combinations of DAAs. The INFORM-1 trial combined R7128 and an HCV protease inhibitor (RG7227) in the absence of IFN-α and ribavirin. Treatment naïve, treatment experienced and null responders receiving this combination for a 14-day period experienced median reductions in HCV RNA of −4.0log_10_ to −5.1log_10_ in the highest dose treatment groups with 25% of patients having viral loads below the limit of detection on the 14th day of treatment. No serious adverse effects were reported [58]. No resistance mutations were identified after four weeks of treatment in patients treated with this combination, although insertion of the S282T mutation into NS5B resulted in a 3–7 fold reduction in sensitivity to R7128 in cell culture [59]. These studies are ongoing with the INFORM-2 trial to look at a combination of the protease and polymerase inhibitors over a four week period in patients infected with genotype 1 virus who previously failed treatment.

4′-azidocytidine (R1479) also entered clinical development. This NI appears to have multiple mechanisms of action. During the initiation phase, the incorporated inhibitor acts as a chain-terminator [60]. Once the enzyme enters the elongation mode, the NI no longer acts as a chain terminator but instead as a base-pair confounder [43]. The enzyme undergoes significant conformational changes during transition from initiation to elongation, which may explain why the inhibitor appears to act by two distinct mechanisms. Treatment of HCV replicon-containing cells with this inhibitor selects for the S96T mutation, although the mechanism of resistance has yet to be elucidated [61]. The S96 residue is in proximity to the bound template at the active site (Figure 1) and it is conceivable that the mutation affects the interaction with the nucleic acid substrate. Like PSI6130, both cytidine and uridine derivatives of this compound were developed. While 4′-azidocytidine was effective in cell culture, 4′-azidouridine was not [62]. When it was delivered as a phosphoramidate prodrug and bypassed phosphorylation to the monophosphate, however, it efficiently inhibited the virus in cell culture [62]. The prodrug of 4′-azidocytidine (R1626) was tested in genotype 1 infected, treatment naïve patients in combination with IFN-α and/or ribavirin. −3.1log_10_ to −4.6log_10_ reductions of HCV RNA were seen in the different treatment arms with triple combination therapy giving the greatest reduction [63]. No selection of resistance mutations was noted during treatment, indicating that, as with other NIs, there is a high barrier to the development of resistance. During treatment, neutropenia and anemia were reported as dose-dependent side effects [63]. Development of R1626 has been placed on hold at this point due to toxicity issues.

Acyclic nucleoside phosphonates compete with natural nucleotides for incorporation and act as chain terminators. These inhibitors are active against a broad range of DNA viruses and presently efforts are underway to target the HCV polymerase [64]. Alkoxyalkyl esters of the compounds have been shown to inhibit HCV replication *in vitro* [65]. Treatment of HCV replicon-containing cells for 6–8 weeks with octadecyloxyethyl 3-methoxy-2-(phosphonomethoxypropyl) adenine (ODE-S-MPMPA) selects for mutations within or in proximity to the NTP tunnel. The active site mutation S282T also leads to an increased EC_50_ with this compound [66].

The strict conservation of the NS5B active site residues makes NIs promising drug candidates to target the polymerase irrespective of the genotype. One of the limitations of NIs is the need for phosphorylation by cellular kinases in order to be used as substrates, since delivery of triphosphates across the cell membrane is inefficient. Delivery of NIs in prodrug form, however, has helped overcome the issue of poor phosphorylation that limits the effectiveness of certain NIs in cell culture. Another potential limitation to this class of inhibitors is the high concentration of competing NTPs in the cell, which are present in the high micro to low millimolar range [67]. Competition with NTPs is accentuated by the higher affinity with which NTPs bind at the enzyme active site as compared to NIs [68].

### 3.2. Non-Nucleoside Inhibitors

NNIs provide an alternative mechanism to target viral polymerases. HIV non-nucleoside reverse transcriptase inhibitors are approved for clinical use and target a single non-substrate binding site on HIV-1 RT [35]. Of note, these inhibitors are not active against HIV-2, which shows natural resistance to these compounds. NNIs targeting NS5B are in development. Greater variability is possible with HCV inhibitors as multiple allosteric binding sites are present on the HCV polymerase. Four binding sites have been identified: NNI site I and site II are located in the thumb domain, while III and IV are closer to the active site in the palm domain.

### 3.3. NNI Binding Sites

#### 3.3.1. NNI I

##### Benzimidazoles and Indoles

**Figure 5:**
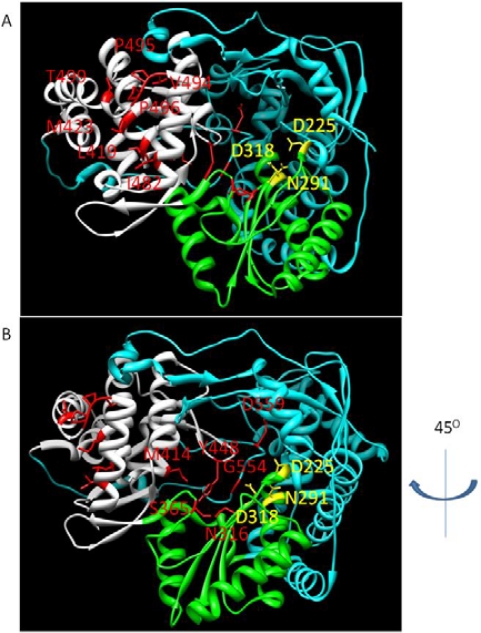
NNI binding sites with key resistance mutations highlighted. Active site residues are labeled in yellow. Domains are colored as follows: thumb, white; fingers, cyan; palm, green. A: highlights the positions of NNI site I and II resistance; B: is a 45 degree rotation of the structure, highlighting sites for resistance to NNI site III and IV inhibitors.

NNI site I inhibitors target a site on the upper section of the thumb domain, approximately 30Å from the active site at the juncture of the thumb and finger loop, a small section of the fingers domain that extends to interact with the thumb domain [69]. Activity of benzimidazole-based compounds in cell culture shows high potency and selectivity towards the NS5B polymerase [70]. Resistance to these compounds arises though the amino acid substitutions P495S/A/L, P496S/A and V499A. Positioning of selected NNI site I mutations, as well as other positions of NNI resistance, is shown in Figure 5.

Substitutions at P495 confer the highest level of resistance, followed by P496 and V499A with only low-level resistance [69]. Resistance is mediated through a lowered affinity for the inhibitor [31]. Indole-based inhibitors also target NNI site I. A crystal structure of NS5B co-crystallized with an indole-based inhibitor revealed that binding appears to displace a section of the finger loop anchoring the region to the thumb domain [71]. It is hypothesized that the α-helix finger loop region is flexible and a semi-open conformation would allow binding of the inhibitor. Subsequently, the bound inhibitor would interfere with conformational changes required to form a productive RNA/enzyme complex and enter into the elongation stage, inhibiting elongation [71].

Testing of an NNI site I inhibitor, BILB-1941, in genotype 1, chronically infected HCV patients demonstrated the antiviral activity of this class of compounds. The effects were subtype specific, with genotype 1a infected patients showing significantly lower response rates than genotype 1b infected individuals [72]. Also targeting NNI site 1 is BI207127, which shows antiviral activity in cell culture against genotype 1a and 1b virus. This NNI has been tested in combination with the standard of care in treatment naïve and treatment experienced patients. The treatment naïve group all showed greater than a −3.0log_10_ drop in viral RNA with no viral rebound after four weeks of therapy. Genotype 1a and 1b patients had similar response rates. In contrast, in the treatment experienced group genotype 1b patients responded better than 1a patients and viral rebound was observed in some individuals in this group [73]. Testing of BI207127 in combination with a protease inhibitor (BI201335) and ribavirin is underway. A third site I inhibitor, MK3281, is active against both genotype 1 and 3 in cell culture. Oral dosing of this inhibitor in chimpanzees led to a −1.4log_10_ drop in viral load after 48 hours in a genotype 1a infected chimpanzee at the highest dose while in a genotype 1b infected animal a −3.8log_10_ drop was observed at the same dosing concentrations [74]. Clinical testing of monotherapy with MK3281 for seven days showed the highest drop in viral load in genotype 1b patients, none of which experienced viral breakthrough during treatment. In contrast, genotype 1a and three infected patients saw lower drops in viral load and viral rebound during treatment was observed in these groups [75].

#### 3.3.2. NNI II

##### Thiophenes, Phenylalanines, Hydroxypyranones, and Pyranoindoles

The binding pocket of the NNI site II inhibitors is a shallow hydrophobic pocket situated at the base of the thumb domain [76]. Thiophene-2-carboxylic acids have been reported as inhibitors of the NS5B polymerase [77,78]. These bind in the thumb domain approximately 35Å from the active site. As with NNI site I inhibitors, it appears that these inhibitors interfere with the interaction between the fingers and thumb domain, preventing the polymerase from adopting the closed conformation required for productive polymerization during elongation [79]. Treatment of HCV replicon-containing cells with this class of compounds selects for the resistance mutations L419M, M423T/I and I482L [80]. The mechanism of resistance is via a reduced affinity for the inhibitor in the presence of the mutations [80]. Filibuvir is one site II NNI undergoing clinical testing. Combining the inhibitor with IFN-α and ribavirin led to marked increases in the rate of achieving a rapid virological response (undetectable HCV RNA four weeks into treatment) and was well-tolerated [81]. Currently, progress beyond week 4 is being followed. A second NNI site II inhibitor, VCH759, is a thiophene-2-carboxylic acid derivative active against genotype 1a and 1b. When given to genotype 1 patients as a monotherapy for 14 days the drug was well-tolerated and viral load decreases at the highest dose were −2log_10_ or greater. A genotype 6 infected individual experienced no decline in viral load. Viral breakthrough during treatment was associated with resistance mutations at the M423, L419, I482 and V494 positions [82].

#### 3.3.3. NNI III

##### Benzothiadiazines and Acylpyrrolidines

NNI III inhibitors bind at a site on the inner thumb/palm domain, close to the active site. Benzothiadiazine-based inhibitors show low nM potency in the replicon system, though the effects appeared to be genotype specific with strong activity against genotypes 1a, 1b and 2a but much lower activity against genotype 3a [83]. Similar to other allosteric inhibitors, these compounds inhibit the enzyme prior to RNA elongation [31,32]. Treatment of cells containing the HCV replicon with these inhibitors selects for various resistance mutations including H95Q, M414T, C451R and G558R [30]. ANA598, a benzothiadiazine-based inhibitor, was given to treatment naïve patients for three days. Patients experienced a median drop in viral RNA of −2.4log_10_ with genotype 1b, which was a higher drop than that seen with 1a [84]. A second study combining the inhibitor with the standard of care saw patients reach undetectable viral levels at earlier points than with the standard of care alone [85]. Resistance to ANA598 is through substitutions at positions C316, M414, Y448 and G554 [85]. A second class of inhibitors targeting NNI site III are the acylpyrrolidines. The binding site of this class of compounds was determined through co-crystallization with NS5B [86]. GSK625433 is an acylpyrrolidine with activity against genotype 1a and 1b described in cell culture [87]. I447F and M414T are the major mutations associated with resistance to this compound. In the replicon system, no cross-resistance was observed with other classes of polymerase inhibitors and the inhibitor was found to act in a synergistic manner with IFN-α [87].

#### 3.3.4. NNI IV

##### Benzofurans

The second palm domain binding site resides in a large hydrophobic pocket. The site is targeted by benzofuran inhibitors. Resistance mutations selected by treatment with a benzofuran inhibitor, HCV-796, include C316N/Y and S365T. The C316Y mutation appears to interfere with binding of the inhibitor to NS5B via a steric clash [88]. Ser365 is required for hydrogen bonding between a side-chain oxygen and the amide nitrogen on HCV-796, and mutations likely disrupt this interaction [89]. Replicon cells containing mutations conferring resistance to this compound also showed resistance to an anthranilate compound which binds at a different NNI site [89,90]. This result suggests that there may be overlap between the two binding sites and cross-resistance could be an issue [89]. Resistance selection occurred rapidly in the HCV replicon system during treatment with HCV-796. After 3 days, the C316Y mutation was detected in both genotype 1a and 1b replicons [91]. This mutation was also detected in patients who experienced viral rebound during clinical testing with HCV-796. Development of the compound was placed on hold after elevated liver enzymes were detected in some patients during phase II clinical trials [89]. ABT-333 is also active against genotype 1a and 1b. Two days of monotherapy with the drug followed by 26 days in combination with the standard of care led to average viral load decreases of −3.7log_10_ in all dosing groups. *In vitro* studies with ABT-333 identified C316Y, M414T, Y448H, S556G and D559G as the major mutations conferring resistance to the compound. As with other NNIs, variation in the viral genome may result in pre-existing resistance mutations. In this study, it was determined at baseline that already 6% of clones contained resistant variants [92].

The multiple allosteric binding sites on NS5B present an opportunity for development of several different types of NNIs and NIs to target the polymerase in the absence of competition. The location of NNI binding sites distant from the active site may also lower the barrier for the development of resistance towards NNIs. The general lack of cross-resistance for NNIs from different binding sites, however, could be exploited during treatment in cases where resistance mutations for one site are already present in the viral genome. Another limitation of NNIs, however, is their genotype and even subtype specificity. Most NNIs in development have been optimized to target genotype 1 virus, and activity against other genotypes is often decreased or absent. This problem is reminiscent of non-nucleoside reverse transcriptase inhibitors (NNRTIs) targeting specifically HIV-1 and not HIV-2.

### 3.4. Pyrophosphate Analogues

The PPi analogue foscarnet and related compounds have been shown to be active against HIV, cytomegalovirus and herpes virus [34]. Toxicity and poor bioavailability, however, have limited their use in a clinical setting except during salvage therapy [93]. Derivatives of 4,5-dihydroxypyrimidine carboxylic acid and α, γ-diketo acids have been developed to target the HCV polymerase. These inhibitors bind the divalent metal ions at the active site of NS5B in a fashion mimicking PPi binding [33,94,95]. PPi released during the nucleotidyl transfer reaction binds transiently at the site previously occupied by the β- and γ-phosphate of the incoming nucleotide triphosphate. This positioning permits the nucleophilic attack of the newly released PPi on the terminal phosphodiester bond, reversing the incorporation reaction. The weak binding of PPi at the active site, however, likely precludes this occurrence during RNA synthesis [96]. The binding conformation has been exploited to develop analogues that interfere with viral genome replication. The mechanism occurs via competition with nucleotides for binding at the active site, since the inhibitors are competitive with respect to both PPi and nucleotides [97–99]. Treatment of HCV replicon-containing cells with diketo acids selects for resistance mutations at positions G152 and P156. These mutations also confer resistance to dihydroxypyrimidine carboxylates [99]. Unlike NNIs, PPi analogues have been reported to be effective at both the initiation and elongation stages of RNA synthesis, again presumably through occupation of the NTP binding site [98]. These inhibitors may also act by interfering with excision of incorporated nucleoside analogues through their competition with PPi [97]. PPi analogues have yet to progress into clinical development.

## 4. Antiviral Resistance

The development of resistance is dependent on a number of factors. Amino acid substitutions that occur through a single nucleotide change will be more easily formed than those requiring multiple nucleotide changes. A clear example of this is seen during treatment with the protease inhibitor teleprevir [100]. This inhibitor selects for the R155K mutation. To generate the resistance mutation, a single nucleotide change is required in genotype 1a infected individuals (AGG to AAG), while two changes are required in genotype 1b infected individuals (CGG to AAG). The mutation appears frequently during treatment of patients with subtype 1a, but not in those with subtype 1b [101].

The comparison of NNIs and NIs suggests that the capacity to support amino acid substitutions at the active site is much lower than at allosteric sites which exist at the surface of the enzyme and can support amino acid changes that do not disrupt the function of the polymerase. In patients, monotherapy with the NNI HCV-796 selected for viral variants containing resistance mutations during the 10 day treatment period in the majority of patients [102]. In contrast, treatment with the NI R1626 for a 14 day period failed to generate resistance [103].

The selection of variants is also dependent on the level of drug resistance achieved with each resistance mutation as well as the replicative capacity of the variant. The S96T mutation, selected by the NI R1479, leads to a 3-fold loss of sensitivity towards the inhibitor and a 96% loss of activity of the enzyme [104]. The S282T mutation, conferring resistance to 2′-*C*-methylated NIs such as PSI6130 and PSI7851/PSI7977, shows a moderate loss of sensitivity to the inhibitors (3–24 fold) but shows an 85% loss of replication capacity, which may account for the delayed kinetics of appearance [54,104,105]. Selection of resistance with the NNI HCV-796 also illustrates the potential for replication capacity to influence whether certain substitutions are selected. Treatment with the inhibitor can select for the mutations C316Y or S365T. The C316Y mutation leads to a 100-fold reduction in sensitivity to the inhibitor, while the S365T mutation lowers sensitivity 500-fold. The replicative capacity of the S365T mutant is 10-fold lower than that of C316Y, and it was not selected during a 3-week assay in replicon cells, despite the higher level of resistance it confers. This is potentially due to its reduced ability to replicate as compared to C316Y [104].

## 5. Pre-Existing Mutations

Considering the estimated error rate of the polymerase, it was recently predicted that within an infected individual all mutants with single or double nucleotide substitutions would be generated multiple times daily [12]. Most mutations, however, are lethal or are not replication fit and these are eliminated from the population [106]. In the absence of drug selective pressure, variants containing polymorphisms at sites for drug resistance may exist as the dominant species, leading the consensus sequence for certain genotypes to contain reported drug-resistance mutations [107]. This has been reported for both HCV protease and polymerase inhibitors. In the case of the protease, treatment naïve subjects with genotype 1 infection showed the V36M resistance mutation in 0.9% of patients, which leads to low-level resistance to the protease inhibitor teleprevir. Also, 0.7% have the R155K mutation that confers high level resistance to the protease inhibitors BILN 2061 and ITMN-191 [108]. Another protease inhibitor resistance mutation, A156T was identified in approximately 1% of patients [109]. Studies on the polymerase have similarly identified genetic variation that confers resistance to certain polymerase inhibitors. M423V and M423I, which confer resistance to certain NNIs, were present in 2.8% of genotype 1a infected patients. In contrast, NI resistance mutations such as S96T and S282T were not identified [110]. M414T has also been identified in treatment naïve patients, as well as at low levels in the HCV replicon system. These polymorphisms are problematic for treatment, since their selection can lead to rapid resistance. For example, treatment of HCV replicon cells with a quinolone benzothiadiazine inhibitor selects for the M414T mutation. This mutation was shown to be present at low levels in the replicon system as compared to the dominant sequence but is selected very rapidly during treatment with levels of the resistant replicon peaking at three days [111]. Immune-selective pressure can also play a role in this occurrence. For example, host human leukocyte antigen-restricted epitopes, such as A24 and B27, overlap with NS5B amino acids 419, 423 and 451. Polymorphisms at these positions conferring resistance to NNIs may be selected over the wild type sequence during immune-driven selection [107]. Combination therapy that suppresses viral replication to a maximum is therefore inevitable in attempts to minimize the risk of resistance development [112].

## 6. Overcoming Resistance

Since HCV monotherapy is strongly associated with emergence of resistance mutations, approaches that combine multiple inhibitors, *i.e.* multiple direct-acting antivirals (perhaps in the absence of the standard of care) with unique resistance profiles will be required for treatment.

The barrier to resistance for classes of inhibitors currently in clinical development for HCV appears to be lowest for NNIs, followed by protease inhibitors, then NIs [113]. Inhibitors of NS5A appear to show a low barrier of resistance; however, the intrinsic potency of these compounds is extremely high [114]. Inhibitors of the cyclophilin A, *i.e.* a cellular factor involved in HCV replication, have been associated with resistance mutations in NS5A and NS5B. In this case, the barrier to the development of resistance appears to be high and the amino acid changes involved appear to confer only low levels of resistance. The development of STAT-C inhibitors is advancing rapidly, with many viral and host proteins now targeted for drug development (reviewed in [115]). Combining different classes of inhibitors that show no cross-resistance can delay the kinetics of the appearance of resistance. For example, combining a polymerase and protease inhibitor (RG7128 and RG7227) resulted in no antiviral resistance in patients after four weeks of treatment, even though protease inhibitors have a relatively low genetic barrier to resistance [59]. Within the NNIs it may also be possible to combine inhibitors, since allosteric inhibitors binding to the different sites on NS5B generally do not show cross resistance. This approach must be used with caution, however, since treatment with two types of allosteric inhibitors has been shown to select for variants containing two distinct resistance mutations [80]. In this study, combination of allosteric inhibitors binding in the thumb and palm domains selected a small number of replicons containing both the M414T and M423T mutations, though the frequency of selecting the double mutant was estimated at 0.006% as compared to 1.3% with a single inhibitor [80].

## 7. Conclusions and Perspectives

NIs and NNI that are designed to target HCV NS5B have distinct biochemical properties, which translates into different clinical challenges and opportunities. The pan-genotype activity associated with NIs is a major plus for this class of compounds. Antiviral activity against a broad spectrum of genotypes suggests that the nucleotide binding site is relatively conserved, and subtle structural variations that may exist in this region are not sufficient to cause significant changes in drug susceptibility. A conserved nucleotide binding site may also help to explain the apparent restrictions in the selection of resistance conferring mutations associated with NIs. Only two amino acid changes, namely S282T and S96T, have been identified and confirmed. Moreover, while the S282T mutation resides in close proximity to the active site, the S96T mutation is located at a distant position. However, it remains to be seen whether the limited number of mutations is indeed related to intolerable reductions in viral fitness of other variants, or merely a consequence of the rather limited structural diversity of nucleoside analogues that have been studied so far.

In HIV, numerous nucleotide reverse transcriptase inhibitor (NRTI) associated mutations, that are clustered around the active site, have been identified [116]. These changes discriminate against the inhibitor through various mechanisms based on differences in inhibitor binding and/or incorporation. Another major group of mutations, referred to as thymidine analogue associated mutations, are located further away from the active site and support binding of the pyrophosphate (PPi) analogue ATP, which, in turn, facilitates excision of incorporated nucleotide analogues. Such mechanistic diversity has not yet been reported for NIs that target NS5B. Thus, it will be interesting to see in the future whether an increase in chemical diversity of investigational NIs may also be associated with an increase in the variety of resistance conferring mutations. The intrinsic ability of HCV NS5B to excise incorporated chain-terminators has been documented, and this reaction may therefore compromise the activity of certain NIs in the absence of amino acid changes [38]. In this context, it is perhaps important to note that purines are less efficiently excised then pyrimidines, which has been documented for HIV-1 RT and HCV NS5B [38,117,118].

A potential challenge associated with the use of NIs is that the triphosphate form of these compounds has to compete with high intracellular concentrations of natural NTPs. There is a fundamental difference between NIs that target RNA polymerases and NRTIs or other nucleoside analogue inhibitors that interfere with viral DNA polymerases. Intracellular concentrations of NTP pools are found to be in the high μM to low mM range, while intracellular concentrations of dNTPs are in the low μM range [119]. Thus, competition at the triphosphate level is by comparison much more unfavorable with nucleotides that target RNA polymerases. As a consequence, the risk of toxic side effects may increase with high concentrations of drug required to overcome this barrier. Intracellular concentrations of ATP are usually higher than intracellular concentrations of GTP and UTP, followed by CTP having the lowest intracellular concentration, although the actual concentration of NTPs available for the viral polymerase is not clear as cellular compartmentation may limit access to NTP pools [67]. This suggests that the therapeutic window of pyrimidine-based inhibitors may be superior in some cases [119]. However, side-by-side comparisons *in vivo* are not available at this point. Moreover, this issue is complicated by findings showing opposite trends with respect to other biochemical properties, including the efficiency of excision [38]. Other important parameters that are not discussed in detail in the review, including the kinetics that characterize build-up and decay of intracellular triphosphate concentrations of the inhibitor, will also impact on its overall performance.

NNIs do not require metabolic activation and these compounds do not appear to compete with intracellular nucleotide pools. Thus, there is no obvious or predictable toxicity issue associated with these compounds. Several independent studies suggest that NNIs may affect the interaction with the nucleic acid substrate, although the detailed nature of the mechanism of action needs to be elucidated. At this point, it is also not clear how the different classes of NNIs exert their inhibitory effects. The binding sites of these compounds do not point to obvious overlaps with the RNA binding channel. Although attempts have been made to develop structural models, it remains to be seen how putative structural perturbations upon NNI binding translate in RNA substrate binding during initiation. Structures of ternary complexes with NS5B, primer/template and inhibitor are not available—neither for HCV NS5B nor for HIV-1 RT. However, detailed kinetic analyses suggest that NNRTIs affect the chemical step during elongation, which appears to distinguish these compounds from HCV-specific NNIs.

NNRTIs and HCV-specific NNIs select rapidly for resistance *in vitro* and *in vivo*, which points to structural flexibilities in the vicinity of inhibitor binding sites. A considerable distance to the active site is a prerequisite for such plasticity. New generation NNRTIs decrease the risk of resistance development through increased structural flexibility of the inhibitor [120], and it will be interesting to see whether a similar strategy can be applied to NNIs. However, the low barrier to the development of resistance and genotype specificity remain a challenge at this point. Combinations of DAAs, in the absence of cross-resistance, will almost certainly reduce the risk of resistance development. It will be of great interest to see whether certain combinations of drug classes perform better than others in this regard. Complementary mechanisms of inhibition associated with different NNIs, or even with different NIs that differ in their base moiety, or combinations of NNIs and NIs may lead to synergistic effects. The advanced development of protease inhibitors, cyclophilin A and/or NS5A inhibitors opens even more potentially promising alternatives in the future.

## Figures and Tables

**Table 1:** Clinical progress of HCV NS5B nucleoside and non-nucleoside inhibitors.

**Inhibitor**	**Company**	**Target**	**Progress**
NM283	Idenix/Novartis	Active site	Stopped
R7128	Roche/Pharmasset	Active site	Phase II
IDX184	Idenix	Active site	Phase II
R1626	Roche/Pharmasset	Active site	Stopped
PSI7851	Pharmasset	Active site	Phase II
MK0608	Merck	Active site	Unknown
HCV-796	ViroPharma/Wyeth	NNI IV	Stopped
PF-868554, Filibuvir	Pfizer	NNI II	Phase II
ABT-333	Abbott	NNI IV	Phase II
ABT-072	Abbott	NNI IV	Phase II
VCH-759	Vertex	NNI II	Phase II
VCH-916	Vertex	NNI II	Phase II
BILB-1941	Boehringer Ingelheim	NNI I	Stopped
IDX375	Idenix	Palm	Phase I
GS9190	Gilead	NNI IV	Phase II
MK-3281	Merck	NNI I	Phase I
BI207127	Boehringer Ingelheim	NNI I	Phase I
VCH-222	Vertex	NNI II	Phase II
ANA598	Anadys	NNI III	Phase II
JTK-109	Japan Tobacco	NNI I	Stopped
GSK625433	GlaxoSmithKline	NNI III	Phase I
